# Should we breathe quiet or noisy?

**DOI:** 10.1186/cc13762

**Published:** 2014-03-11

**Authors:** Christian Putensen, Thomas Muders

**Affiliations:** 1 Department of Anesthesiology and Intensive Care Medicine, University of Bonn, Bonn, 53105, Germany

## Abstract

External noise is introduced by computer-generated random levels of pressure assistance during noisy pressure support ventilation (PSV). In patients, noisy PSV was associated with higher tidal volume variability but not improved cardio-pulmonary function compared with conventional PSV. The potential role of noisy PSV in the management of critically ill patients requiring ventilatory support has to be explored further.

## 

Although introduced as weaning techniques, modes providing mechanical support of spontaneous breathing have become standard in primary mechanical ventilator support in critically ill patients. In *Critical Care*, Spieth and colleagues [1] report for the first time the use of noisy pressure support ventilation (PSV) in patients with acute hypoxemic respiratory failure.

Normal breathing shows considerable variation in tidal volume (V_T_), flow rate, and respiratory rate, which is lost during mechanical ventilation (MV). In addition to elevation of intrathoracic and intrapulmonary pressures, MV causes a non-physiological uniform breathing pattern. Based on the concept that MV should mimic physiologically noisy breathing patterns, biologically variable or noisy MV was introduced, which attempted to mimic spontaneous breath-to-breath variability during volume-controlled MV [2]. Experimental and small clinical trials suggest that biologically variable or noisy MV may improve pulmonary gas exchange, compliance and dead space by preventing de-recruitment when compared to conventional MV [2,3]. These findings support the concept that alveolar recruitment achieved by large V_T_ exceeds the de-recruitment by small V_T_. Other mechanisms claimed to explain improved lung function during biologically variable or noisy MV include stochastic resonance [4], increased respiratory sinus arrhythmia [5], endogenous surfactant release [6,7], and dynamic effects on the pressure-volume curve [7].

Nowadays, modes providing assisted support of spontaneous breathing should not only assure adequate gas exchange and unloading of the patient’s work of breathing but should provide patient-ventilator synchrony, optimized diaphragmatic unloading, lung-protective ventilation, and the preservation of physiological respiratory patterns and variability. To accomplish all these tasks ventilatory assistance can no longer be constant but has to continuously adapt to changes in ventilatory demand and respiratory mechanics. To adapt to the continuously changing respiratory demand, pressure assistance is proportional to the instantaneous flow and volume, reflecting the inspiratory muscles’ pressure during proportional assist ventilation (PAV) [8] or proportional to the electrical activity of the diaphragm during neurally adjusted ventilator assist (NAVA) [9]. Thus, PAV and NAVA try to amplify the patient's respiratory center output. Several experimental investigations and clinical trials in small groups of critically ill patients have demonstrated that PAV and NAVA, when compared to conventional PSV, enhance patient-ventilator interaction and synchrony, which translates into better comfort and sleep quality and preserves V_T_ variability, which has been associated with improvements in gas exchange and lung mechanics [10,11].

In patients with mechanically assisted spontaneous breathing, the noise can be introduced externally or can come directly from the respiratory center. In contrast to PAV and NAVA, which amplify the noise coming from the respiratory center, external noise is introduced by computer-generated random levels of pressure assistance during noisy PSV [12]. Experimental investigations in induced lung injury showed that noisy PSV, when compared with conventional PSV and pressure-controlled MV, was associated with a significantly higher coefficient of variation of V_T_ and airway pressure, and resulted in better pulmonary gas exchange, reduced alveolar edema in overall lung as well as reduced inflammation in the nondependent parts of the lungs [13]. In a porcine model with induced lung injury an external source of noise (noisy PSV) and noise derived from amplification of the respiratory center (PAV) improved gas exchange and produced higher V_T_ variability, whereas the pulmonary inflammatory response and diffuse alveolar damage score did not differ when compared to conventional PSV lungs [14].

Spieth and colleagues [1] investigated the short-term effects of conventional and noisy PSV in patients with acute hypoxemic respiratory failure. Noisy PSV was associated with higher V_T_ variability and a lower number of asynchrony events. In contrast to experimental findings, cardio-pulmonary function and spatial distribution of ventilation were comparable between conventional and noisy PSV [1]. During conventional and noisy PSV, however, V_T_ significantly higher than 8 ml/kg predicted body weight was frequently noticed. By design noisy PSV applies V_T_ as high as 16 ml/kg and as low as 1.6 ml/kg once every 20 to 30 minutes [12]. Although short-term experiments demonstrate that this mixture of ultra-protective and non-protective V_T_ during noisy PSV does not add to lung injury based on histological examinations, long-term investigations have to clarify the relevance of periodic non-protective V_T_ ventilation in critically ill patients.

## Conclusion

The findings of Spieth and colleagues [1] are in line with the observation that, in patients with acute hypoxemic respiratory failure, PAV was associated with higher V_T_ variability but did not improve cardio-pulmonary function when compared with conventional PSV [10]. Thus, application of an external source of noise with noisy PSV and amplification of the respiratory center-derived source of noise with PAV may both allow higher V_T_ variability [1,10]. However, we have to question if higher V_T_ variability improves outcome, reduces cost, decreases the frequency of complications, or simplifies patient management or care giving. Unfortunately, apart from experiments or small clinical trials showing some physiological or clinical benefits, noisy PSV must still show its real clinical benefits in large clinical trials before becoming a routine method of ventilation. Thus, noisy ventilation is clearly not something that should be currently adopted in either controlled or assisted MV.

## Abbreviations

MV: Mechanical ventilation; NAVA: Neurally adjusted ventilator assist; PAV: Proportional assist ventilation; PSV: Pressure support ventilation; VT: Tidal volume.

## Competing interests

The authors declare that they have no competing interests.
